# Smart Shirts for Monitoring Physiological Parameters: Scoping Review

**DOI:** 10.2196/18092

**Published:** 2020-05-27

**Authors:** Hamzeh Khundaqji, Wayne Hing, James Furness, Mike Climstein

**Affiliations:** 1 Faculty of Health Sciences & Medicine Bond University Gold Coast Australia; 2 School of Health and Human Sciences Southern Cross University Bilinga Australia; 3 Physical Activity, Lifestyle, Ageing and Wellbeing Faculty Research Group University of Sydney Sydney Australia

**Keywords:** wearable electronic devices, biomedical technology, telemedicine, fitness trackers, sports, exercise, physiology, clinical decision making, vital signs

## Abstract

**Background:**

The recent trends of technological innovation and widescale digitization as potential solutions to challenges in health care, sports, and emergency service operations have led to the conception of smart textile technology. In health care, these smart textile systems present the potential to aid preventative medicine and early diagnosis through continuous, noninvasive tracking of physical and mental health while promoting proactive involvement of patients in their medical management. In areas such as sports and emergency response, the potential to provide comprehensive and simultaneous physiological insights across multiple body systems is promising. However, it is currently unclear what type of evidence exists surrounding the use of smart textiles for the monitoring of physiological outcome measures across different settings.

**Objective:**

This scoping review aimed to systematically survey the existing body of scientific literature surrounding smart textiles in their most prevalent form, the smart shirt, for monitoring physiological outcome measures.

**Methods:**

A total of 5 electronic bibliographic databases were systematically searched (Ovid Medical Literature Analysis and Retrieval System Online, Excerpta Medica database, Scopus, Cumulative Index to Nursing and Allied Health Literature, and SPORTDiscus). Publications from the inception of the database to June 24, 2019 were reviewed. Nonindexed literature relevant to this review was also systematically searched. The results were then collated, summarized, and reported.

**Results:**

Following the removal of duplicates, 7871 citations were identified. On the basis of title and abstract screening, 7632 citations were excluded, whereas 239 were retrieved and assessed for eligibility. Of these, 101 citations were included in the final analysis. Included studies were categorized into four themes: (1) prototype design, (2) validation, (3) observational, and (4) reviews. Among the 101 analyzed studies, prototype design was the most prevalent theme (50/101, 49.5%), followed by validation (29/101, 28.7%), observational studies (21/101, 20.8%), and reviews (1/101, 0.1%). Presented prototype designs ranged from those capable of monitoring one physiological metric to those capable of monitoring several simultaneously. In 29 validation studies, 16 distinct smart shirts were validated against reference technology under various conditions and work rates, including rest, submaximal exercise, and maximal exercise. The identified observational studies used smart shirts in clinical, healthy, and occupational populations for aims such as early diagnosis and stress detection. One scoping review was identified, investigating the use of smart shirts for electrocardiograph signal monitoring in cardiac patients.

**Conclusions:**

Although smart shirts have been found to be valid and reliable in the monitoring of specific physiological metrics, results were variable for others, demonstrating the need for further systematic validation. Analysis of the results has also demonstrated gaps in knowledge, such as a considerable lag of validation and observational studies in comparison with prototype design and limited investigation using smart shirts in pediatric, elite sports, and emergency service populations.

## Introduction

### Background

Recent years have seen a marked trend of technological innovation through widescale digitization in the areas of health care, sports, and emergency operation services [1-3]. Propelled by technological progress and motivated by improving quality of care while reducing costs, the immense volume of health data produced today holds promise for aiding in ways such as clinical decision support, disease surveillance, health management, and performance optimization [1,4,5]. With a global shift toward personalized, preventative, and evidence-based models of care, the use of noninvasive monitoring and data analysis to inform clinical practice and training program design is steadily increasing [1,2,6].

Owing to the large volume, velocity, and variety of data produced, there has been a need to adopt advanced and complex technology capable of data collection, storage, and analysis [4]. Among these innovative technologies are wearable systems for physiological metrics tracking conceived by the convergence of microelectronics, wireless communication, and analytics. Designated by the Global Observatory for eHealth as mobile health systems, these forms of technology have been recognized by the World Health Organization as an essential element of electronic health, which prioritizes the cost-effective and secure use of digital technologies in support of medical and public health practice [7,8].

Wearables such as the wrist-worn Fitbit (Fitbit Inc) have garnered incredible commercial acceptance, with revenues reaching US $347 million in the third quarter of 2019 [9,10]. However, despite demonstrating validity, reliability, and acceptability for their estimates of physiological metrics such as heart rate (HR), the use of these devices is largely targeted toward fitness enthusiasts rather than researchers or clinicians [11-13].

Over the past two decades, the increased demand for noninvasive and comfortable long-term tracking of physiological metrics among clinicians has been met through an increase in the research and development of another type of wearable technology known as the smart textile.

Smart textiles are products made up of fibers, filaments, and yarns that host several electronic components, such as sensors, read-out circuits, and embedded communication systems, powered by an integrated or external power supply. Communication systems such as Bluetooth allow for the connectivity of the textile to other intelligent devices for the visualization and analysis of the data obtained in real time. These textile systems, typically designed as electronic-embedded clothing, offer a relaxed structure capable of noninvasive tracking and simultaneous communication of physiological and biomechanical data. In health care, these intelligent textile systems have the ability to support telemedicine and promote the proactive involvement of patients in their medical management through the collection and tracking of their health and diagnostic data [14]. The potential to improve preventative medicine and early diagnosis through the continuous tracking of physical and mental health status as well as physical activity also exists. [14].

In the spheres of sports and emergency response, the ability of these systems to provide continuous physiological data in real time is considerable. The detection and subsequent use of metrics indicative of the physical performance, physiological status, and mental alertness of an athlete or emergency operator have been shown to mitigate injuries and improve performance [2,15]. Although wearable systems such as those produced by Catapult (Catapult Innovations) are currently used by sporting teams to monitor workload and impact, these systems are typically limited in the physiological metrics monitored [6,15,16]. Smart textiles, on the other hand, have the potential to provide medical personnel and performance specialists with additional comprehensive, physiological insights across multiple body systems.

However, it is currently unclear what type of evidence surrounding the use of smart textiles for physiological parameter monitoring exists. For this reason, a scoping review was conducted to systematically survey the existing body of scientific literature on smart textiles in their most prevalent form, the smart shirt, for the monitoring of physiological parameters.

### Objectives

The primary outcomes were to (1) provide a clear indication of the volume and types of scientific literature relating to smart shirts, (2) summarize the studies completed to date, and (3) identify any knowledge gaps to inform future research.

To guide the review, the following research question was formulated: what is the extent, range, and nature of the scientific literature pertaining to smart shirts for physiological monitoring?

## Methods

### Protocol and Registration

An a priori protocol was developed using the Preferred Reporting Items for Systematic Reviews and Meta-analysis (PRISMA) Extension for Scoping Reviews: Checklist and Explanation [17]. The final protocol was registered prospectively with the Open Science Framework (DOI 10.17605/OSF.IO/TNK9X) on August 8, 2019 [18].

### Eligibility Criteria

The eligibility criteria were informed by the Population-Concept-Context framework recommended by the Joanna Briggs Institute (JBI) Reviewer’s Manual [19].

### Population

This scoping review did not impose any restrictions on the population. Men and women of any population or age were suitable for inclusion.

### Concept

The concept of this scoping review was the monitoring of physiological outcome measures using smart textiles in the form of smart shirts. For the purpose of this review, a smart textile was defined as an intelligent textile structure or fabric that possesses integrated sensors for the monitoring and recording of physiological parameters while worn.

### Context

All study designs were considered for this scoping review, which included published articles and reviews, conference proceedings, gray literature, and chapters in the text. Studies conducted across all settings were considered for inclusion. Studies were excluded if they focused on a singular component of the smart shirt (ie, materials, sensors, or algorithms) rather than the smart shirt as an integrated unit. Furthermore, because this review focused on physiological parameters, studies concerning smart shirts for biomechanical or activity monitoring were also excluded.

### Information Sources

To identify potentially relevant literature, a 3-step approach was utilized. First, a limited preliminary search was conducted in 2 electronic bibliographic databases relevant to the topic: Ovid Medical Literature Analysis and Retrieval System Online (MEDLINE) and Excerpta Medica database (EMBASE). The limited search was then followed by analysis of the text words contained in the titles and abstracts of the retrieved papers and of the index terms used to describe them. A second comprehensive search strategy was then developed using all identified keywords and index terms by the lead investigator (HK) in consultation with a librarian highly experienced in electronic searches. Using the final search strategy, the following bibliographic databases were searched from inception of the database to June 24, 2019: Ovid MEDLINE, EMBASE, Scopus, Cumulative Index to Nursing and Allied Health Literature, and SPORTDiscus. The search results were exported into EndNote (Clarivate Analytics), with duplicates removed. Finally, the electronic database search was supplemented by scanning the reference lists of the included studies. The Canadian Agency for Drugs and Technologies in Health (CADTH) gray literature searching tool was also used to identify any nonindexed literature of relevance to this review [20].

### Search

The final search strategy for all databases used can be found in Multimedia Appendix 1. Owing to the large number of irrelevant citations returned by the Scopus database, the search strategy was refined through the inclusion of additional keywords using the “AND” operator to focus the returned results on smart shirts for the monitoring of physiological outcome measures.

### Selection of Sources of Evidence

**Figure 1:**
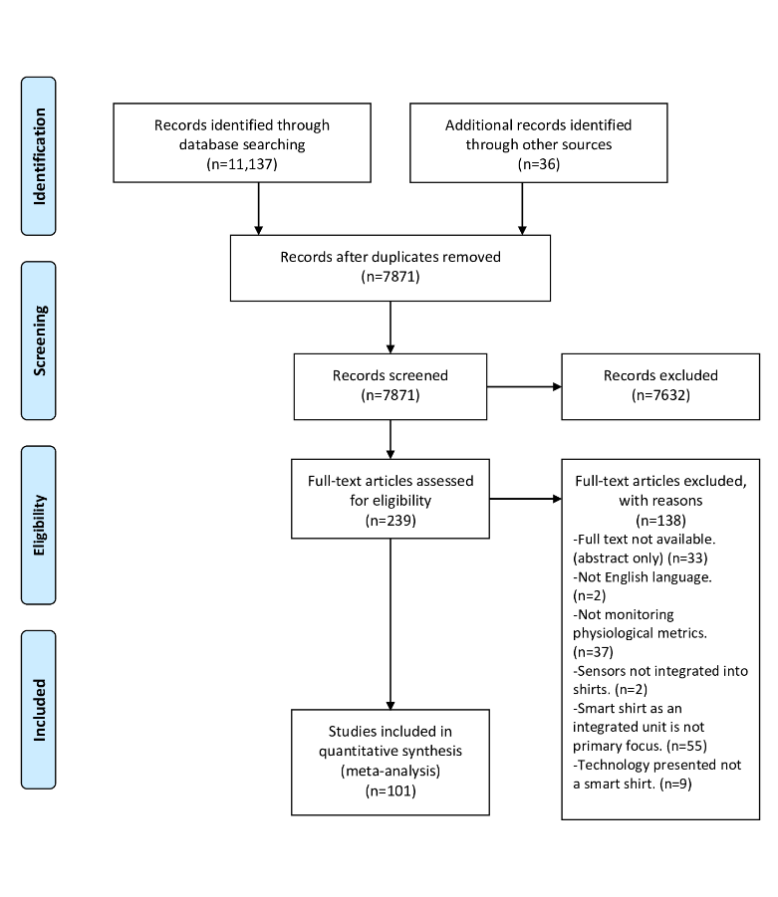
Preferred Reporting Items for Systematic Reviews and Meta-analysis flow diagram. CINAHL: The Cumulative Index to Nursing and Allied Health; EMBASE: Excerpta Medica database; MEDLINE: Medical Literature Analysis and Retrieval System Online.

Using a priori eligibility criteria, a standardized questionnaire for study selection was developed to assist in the screening of titles, abstracts, and full text (Multimedia Appendix 2). A pilot exercise preceded each level of screening. Any queries raised by the pilot exercise were reviewed and resulted in the amendment of the questionnaire by the lead investigator. Following the removal of duplicates, the lead investigator screened papers based on title and abstract. Papers that did not meet the eligibility criteria were removed. Subsequently, the full texts of the remaining papers were retrieved and screened to determine their eligibility. As per the PRISMA guidelines, a flow diagram outlining the study selection process was produced (Figure 1). A critical appraisal of individual sources of evidence was not undertaken because this scoping review aimed to provide a map of the extent, range, and nature of the existing evidence rather than seek the best available evidence related to practice or policy [17].

### Data Charting and Data Items

First, a data-charting form was adapted from the JBI Methodology Guidance for Scoping Reviews at the protocol stage [19] (Multimedia Appendix 3). Main areas of interest were identified, such as study citation details (eg, author, year of publication, reference type, country of origin, and study design), key study characteristics (eg, sample characteristics, study aims, type of smart shirt used, comparators, and outcomes measured), and key findings. Once the form was created, it was tested in a pilot data-charting exercise using 10 studies to ensure that all relevant data were being captured. The data extraction fields were updated through an iterative process that resulted in the inclusion of additional fields for thematic analysis (eg, types of signals acquired, and sensors used). Once the testing was complete, and the data-charting form was refined, the lead investigator (HK) independently screened all included studies and extracted key information from them. Data charted in the final extraction form included study citation details (eg, author, year of publication, reference type, country of origin, and study design), key study characteristics (eg, sample characteristics, study aims, type of smart shirt used, types of signals acquired, types of sensors used, comparators when applicable, and outcomes measured), and key study findings.

### Synthesis of Results

Studies were categorized according to the four main themes identified: (1) prototype design, (2) validation, (3) observational, and (4) reviews. Key study characteristics and findings are graphically represented and tabulated.

## Results

### Selection of Sources of Evidence

Following the removal of duplicates, a total of 7871 citations were identified from searches of the electronic databases, the CADTH gray literature searching tool, and the reference lists of included studies. On the basis of title and abstract screening, 7632 citations were excluded, whereas 239 were retrieved and assessed for eligibility. Of these, 138 were excluded for the following reasons: 33 were abstracts or the author was unable to retrieve the full text, 2 were in Mandarin, 37 were not focused on physiological parameters collected by smart shirts, 2 included technology that failed the study’s definition of a smart shirt due to the lack of integration of their sensors, 55 were focused on only one aspect of a smart shirt (eg, a sensor, materials, or algorithm) rather than the functional unit, and 9 were not smart shirts (eg, chest straps; Figure 1).

### General Study Characteristics

In sorting the included studies by publication type, journal articles were the most prevalent (60/101, 59.4%), followed by conference proceedings (37/101, 36.6%), thesis dissertations (3/101, 3.0%), and reviews (1/101, 1.0%). The years of publication identified in the literature search ranged from 1999 to 2019, with the years 2015 to 2018 producing the majority of publications (47/101, 46.5%). Publications were categorized into four themes of study: (1) prototype design, (2) validation, (3) observational, and (4) reviews. Table 1 presents the general characteristics and associated references of the analyzed publications, including year of publication, type of publication, and theme of study. The countries of origin varied widely, with 24 countries represented by 5 continents: Europe (61/101, 60.4%), North America (18/101, 17.8%), Asia (15/101, 14.9%), Australia and Oceania (4/101, 4.0%), and South America (3/101, 3.0%, Table 2). Among the 24 countries, Italy produced the bulk of the relevant literature (25/101, 24.8%).

Table 2 presents the countries represented within each continent, their respective references, and the number of studies by theme originating from each country.

**Table 1:** Characteristics of included studies (N=101).

Characteristics		Number of studies, n	Reference(s)
**Year of publication**			
	Before 2000	1	[21]
	2000-2004	2	[22]
	2005-2009	25	[3,23-45]
	2010-2014	18	[46-63]
	2015-2018	47	[64-111]
	2019	8	[112-117]
**Type of publication**			
	Journal article	60	[21-23,25,28,32,34-36,39-43,45,46,49,53,56,59-65,67,69-71,73,74,76,78,80-83,85,86,88-90,93,95-97,99-101,104,106,108,109,114-119]
	Conference proceeding	37	[3,24,26,27,29-31,33,37,38,44,47,48,50,54,55,57,58,68,72,75,77,79,84,87,92,94,98,102,103,105,107,110,111,113,120]
	Thesis dissertation	3	[51,66,91]
	Reviews	1	[112]
**Theme of study**			
	Prototype design	50	[3,21,22,24-26,31-34,36-40,42-44,47,50,57-60,67-71,77-80,86-90,99-105,114,118,120]
	Validation	29	[27-29,41,45,46,48,52,53,61-63,72-74,81,92,93,106-111,115-117,119]
	Observational	21	[23,30,35,49,54-56,64,65,75,76,82-85,94,96-98]
	Review	1	[112]

**Table 2:** Countries of origin of the included studies by total and thematic numbers (N=101).

Continent and country of origin		Total number of studies by country, n	Number of studies by theme			
			Prototype design (n=50)	Validation (n=29)	Observational (n=21)	Reviews (n=1)
**Europe**						
	Belgium [32,38]	2	2	N/A^a^	N/A	N/A
	Finland [23]	1	N/A	N/A	1	N/A
	France [27,107]	2	N/A	2	N/A	N/A
	Germany [42,59,60,63,81,98,105]	7	4	2	1	N/A
	Ireland [31,47]	2	2	N/A	N/A	N/A
	Italy [3,25,26,29,30,33,36,37,46,48,49,53,54,56,58,64,76,79,84,92,99,108,110,116,120]	25	10	8	7	N/A
	Poland [95,100]	2	N/A	N/A	2	N/A
	Portugal [68,71,75,80,102,113]	6	6	N/A	N/A	N/A
	Slovakia [57]	1	1	N/A	N/A	N/A
	Spain [39,50,62,101]	4	3	1	N/A	N/A
	Switzerland [28,34,55,72,83,100]	6	2	2	2	N/A
	United Kingdom [45,52,82]	3	N/A	2	1	N/A
**North America**						
	Canada [35,74,87,104,114]	5	3	1	1	N/A
	United States [21,41,61,65,66,73,85,93,96,97,106,109,117]	13	1	8	4	N/A
**Asia**						
	China [40,44,78,88,90]	5	5	N/A	N/A	N/A
	India [43]	1	1	N/A	N/A	N/A
	Japan [70]	1	1	N/A	N/A	N/A
	Malaysia [111]	1	N/A	1	N/A	N/A
	South Korea [22,24,69,89,118,119]	6	5	1	N/A	N/A
	Taiwan [67]	1	1	N/A	N/A	N/A
**Australia**						
	Australia [112]	1	N/A	N/A	N/A	1
	New Zealand [91,94,115]	3	N/A	1	2	N/A
**South America**						
	Chile [77,86,103]	3	3	N/A	N/A	N/A

^a^N/A: Not applicable.

### Study Themes

#### Prototype Design Studies

Throughout the 50 analyzed prototype design studies, the capabilities of the presented smart shirts varied from acquiring one physiological signal (cardiac, respiratory, or surface electromyography [sEMG]) to numerous signals simultaneously (Table 3).

The physiological sensors integrated into the presented prototypes also varied considerably. Across the 50 studies, 10 distinct cardiac and respiratory sensors were identified (Table 4). Among the cardiac sensors, the most prevalent was the textile electrode, which was used in 28 different studies. The piezoresistive sensor, or strain gauge, was the most common respiratory sensor, with its use reported in 10 studies (10/50, 20%). sEMG electrodes were used in all studies (4/50, 8%), presenting a prototype capable of measuring electrical muscle activity. Other physiological sensors integrated into the prototypes included those capable of measuring body temperature (BT) and blood oxygen saturation (SpO_2_). Table 4 presents all the different sensors identified across all prototype design studies.

**Table 3:** Types of signals acquired by the prototypes presented in the included studies (N=50).

Signals acquired	Value, n (%)
Cardiac only [32,57,60,67-69,78,80,88,105,118]	13 (26)
Respiratory only [31,42,47,51,79,87,104]	8 (16)
Electromyography only [38,103]	2 (4)
Numerous signals [3,22,24-26,33,34,36,37,39,40,44,50,58,59,77,80,86,99,102,113,114,120]	27 (54)

**Table 4:** Types and prevalence of the physiological sensors used in prototype studies (categories not exclusive; N=81)

Classification and type of sensor		Number of studies, n	Reference(s)
**Cardiac**			
	Adhesive button electrodes	1	[22]
	Bluetooth heart rate monitor	1	[67]
	Noncontact, metal capacitive electrodes	1	[69]
	Conductive ink electrodes	1	[70]
	Disposable electrodes	1	[24]
	Phonocardiogarphy	1	[44]
	Photoplethysmography	2	[43,44]
	Pulse sensor	2	[100]
	Silicon electrodes	2	[43,114]
	Textile electrodes	28	[3,25,26,32-34,36,37,40,50,57,59,60,68,71,77,78,80,86,88-90,99,102,105,113,118,120]
	Not specified	3	[21,39,58]
**Respiratory**			
	Antenna (fiber, spiral, and hybrid spiral)	2	[51,57,104]
	Fiber Bragg grating sensor	1	[79]
	Impedance pneumography	2	[22,33]
	Noncontact, metal capacitive electrodes	1	[101]
	Optical fiber	1	[42]
	Piezoresistive	10	[3,25,26,33,36,47,77,86,120]
	Polypyrrole	1	[31]
	Respiratory inductive plethysmography	5	[34,37,40,44,114]
	Sensor coil	1	[59]
	Textile	2	[99,102]
	Not specified	1	[58]
**Electromyography**			
	Surface electromyography electrodes	4	[38,102,103,113]
**Oxygen saturation**			
	Pulse oximeter	2	[46,48]
	Not specified	1	[120]
**Body temperature**			
	Monolithic	1	[3]
	Bandgap	1	[50]
	Digital sensor and thermistor	1	[43]
	Not specified	2	[120]

#### Validation Studies

Following prototype design, validation was the most recurrent study theme. All identified studies investigated the validity of a smart shirt against an established reference technology to measure one or more physiological outcome measures*.* A total of 16 types of smart shirts were validated (Table 5). Among these were 3 commercially available shirts, Hexoskin (Carrè Technologies Inc), LifeShirt (LifeShirt VivoMetric), and Zephyr BioHarness (Zephyr Technology), and 13 working prototypes. Included in these prototypes was the Protection e-Textiles system developed as part of a European initiative focused on developing a wearable textile system for emergency operators [46,48]. The physiological sensors integrated into each shirt varied and ranged from combinations of cardiac, respiratory, BT, and SpO_2_ sensors. Table 5 presents the outcome measures validated in each smart shirt.

Table 6 summarizes the physiological sensors integrated in each smart shirt. Multimedia Appendix 4 summarizes the validation studies across citation characteristics, study participants, type of smart shirt used, physiological outcome measures validated, reference technology used as a comparator, and main findings.

**Table 5:** Physiological outcome measures validated by a smart shirt across all validation studies.

Type of smart shirt	Respiratory rate	Minute ventilation	Tidal volume	Breath duration	Heart rate	Electrocardiograph signals^a^	Body temperature	Blood oxygen saturation	Energy expenditure
BioShirt	N/A^b^	N/A	N/A	N/A	N/A	X^c^	N/A	N/A	N/A
GOW system	N/A	N/A	N/A	N/A	N/A	X	N/A	N/A	N/A
HeartCycle’s guided exercise system	X	N/A	N/A	N/A	X	X	N/A	N/A	N/A
Hexoskin	X	X	X	N/A	X	X	N/A	N/A	X
Long Term Medical Survey System	X	N/A	N/A	N/A	X	N/A	X	N/A	N/A
Maglietta Interattiva Computerizzata	X	N/A	N/A	N/A	X	X	N/A	N/A	N/A
Prototype 1 [81]	N/A	N/A	N/A	N/A	X	N/A	N/A	N/A	N/A
Prototype 2 [52]	X	N/A	X	X	N/A	N/A	N/A	N/A	N/A
Prototype 3 [92]	X	N/A	X	N/A	N/A	N/A	N/A	N/A	N/A
Prototype 4 [108]	X	N/A	X	X	N/A	N/A	N/A	N/A	N/A
Prototype 5 [110]	N/A	N/A	N/A	N/A	N/A	N/A	N/A	N/A	N/A
Protection e-Textiles (inner garment)	N/A	N/A	N/A	N/A	N/A	N/A	X	X	N/A
LifeShirt	X	N/A	N/A	N/A	N/A	X	N/A	N/A	N/A
Wealthy system	X	X	N/A	N/A	N/A	N/A	N/A	N/A	N/A
Wearable Wellness System	N/A	N/A	N/A	N/A	N/A	X	N/A	N/A	N/A
Zephyr BioHarnes	N/A	N/A	N/A	N/A	X	N/A	N/A	N/A	N/A

^a^P and T waves, QRS complex, respiratory rate intervals, and heart rate variability.

^b^N/A: not applicable.

^c^X indicates physiological outcome measures validated by studies for the corresponding smart shirt.

**Table 6:** Types of physiological sensors used by smart shirts in the identified validation studies.

Smart shirt and category of sensor		Physiological sensors	Number of studies, n	Reference(s)
**BioShirt**				
	C^a^	1-lead ECG^b^	1	[119]
**GOW**				
	C	1-lead ECG	1	[62]
**HeartCycle’s** **guided exercise**				
	C	1-lead ECG	1	[63]
	R^c^	Not specified	1	[63]
**Hexoskin**				
	C	1-lead ECG	10	[65,73,74,93,106,107,109,111,115,117]
	R	RIP^d^	10	[65,73,74,93,106,107,109,111,115,117]
**LifeShirt**				
	C	2-lead ECG	3	[28,41,45]
	R	RIP	3	[28,41,45]
**Long Term Medical Survey System**				
	C	2-lead ECG	1	[72]
	R	Transthoracic bioimpedance	1	[72]
	SpO_2_^e^	4-channel optical sensor	1	[72]
	BT^f^	BT	1	[72]
**Maglietta Interattiva Computerizzata**				
	C	1-lead ECG	2	[29,53]
	R	Piezoresistive plethysmography	2	[29,53]
**Protection e-Textiles**				
	C	1-lead ECG	2	[46,48]
	R	Piezoresistive plethysmography	2	[46,48]
	SpO_2_	Pulse oximeter (finger)	2	[46,48]
	BT	N/A^g^	2	[46,48]
**Prototype 1**				
	C	12-lead ECG	1	[81]
**Prototype 2**				
	C	1-lead ECG	1	[52]
**Prototype 3**				
	R	FBG^h^	1	[92]
**Prototype 4**				
	R	FBG	1	[108]
**Prototype 5**				
	R	FBG	1	[110]
**Wealthy System**				
	C	5-lead ECG	1	[27]
	R	Impedance pneumography	1	[27]
**Wearable Wellness System**				
	C	1-lead ECG	1	[116]
**Zephyr BioHarness**				
	R	1-lead ECG	1	[61]

^a^C: cardiac.

^b^ECG: electrocardiograph.

^c^R: respiratory.

^d^RIP: respiratory inductance plethysmography.

^e^SpO_2_: blood oxygen saturation.

^f^BT: body temperature.

^g^N/A: not applicable.

^h^FBG: fiber Bragg grating.

#### Observational Studies

Observational studies made up approximately 21.0% (21/101) of the included publications. These studies used smart shirts to capture various physiological outcome measures in a range of populations. In total, 10 types of smart shirts were used, with Hexoskin being the most prevalent (8/21, 38%), followed by the Personalized Monitoring System for Care in Mental Health system (3/21, 14%). The experimental settings varied among the analyzed studies, with the majority (13/21, 62%) being conducted in the field. A total of 5 studies were conducted in a controlled setting such as a laboratory, whereas 2 used both controlled and free-living settings. The populations studied also varied, with the predominant population being clinical (9/21, 43%). The remaining studies used occupational populations such as medical personnel, office employees, firefighters (5/21, 24%), healthy participants (4/21, 19%), and a combination of healthy and clinical participants (3/21, 14%). All studies recruited adults with the exception of one that recruited both adults and pediatric participants [95]. Multimedia Appendix 5 summarizes the observational studies across citation characteristics, type of shirt used, study aim, population characteristics, study setting, and physiological outcome measures tracked.

### Reviews

Only one review focusing on the use of smart shirts for the monitoring of physiological parameters was identified through a literature search. This review was in the form of a scoping review with the objectives of exploring, organizing, and presenting the existing literature on the use of electronic textiles for electrocardiograph (ECG) monitoring in cardiac populations. The review identified resting ECG as the most common form of ECG acquired by electronic textiles, followed by exercise ECG and ambulatory ECG. The primary technical issue reported across all studies was noise from motion artifacts [112].

## Discussion

### Principal Findings

The primary purpose of this scoping review was to systematically analyze the body of scientific literature pertaining to smart shirts for the monitoring of physiological parameters. The primary outcomes were to (1) provide a clear indication of the volume and types of scientific literature relating to smart shirts, (2) summarize the studies completed to date, and (3) identify any knowledge gaps to inform future research.

From the 7871 citations identified after the removal of duplicates, 239 (3.00%) were eligible for full-text review. Of these 239 citations, 101 (1.3%) were included in the final study. Although the percentage of included studies appears small, this was an expected outcome due to the broad search strategy employed. The reason behind the broad search strategy was the lack of standardized terminology in the field of wearables due to its relatively recent inception. For example, smart textiles may be referred to as electronic textiles, e-textiles, electronic devices, wearable devices, wearable monitoring devices, wearable systems, etc. This required the inclusion of a wide range of keywords in the search strategy to maximize the capturing of relevant literature. However, the limitation of this strategy is that the search resulted in many studies that failed the inclusion criteria.

Throughout the screening and review process, four main themes of study were identified: (1) prototype design, (2) validation, (3) observational, and (4) reviews. The most prominent theme was prototype design, accounting for approximately 49.5% (50/101) of the total included studies. These studies presented the design of wearable systems in the form of sensor-integrated shirts for continuous and noninvasive monitoring of cardiorespiratory parameters. Although some prototypes were capable of only monitoring a single parameter (23/50, 46%), the majority (27/50, 54%) could monitor several simultaneously. These physiological parameters were classified as cardiac, respiratory, sEMG, BT, and SpO_2_. Aside from the monitoring capabilities, it was evident that the key focus of design was on the *wearability* of the smart shirts over longer periods. Many of the established technologies used today for the monitoring of physiological parameters such as the ECG or Holter monitor are only capable of short-term diagnostic recording because of their restricted portability and uncomfortable sensors. To circumvent these issues, many of the included studies integrated their smart shirts with textile sensors. Textile sensors are intended to be comfortable, lightweight, flexible, stretchable, conformable, washable, and long lasting. This contrasts with sensors such as the standard single-use, disposable silver-silver chloride (Ag/AgCl) electrodes, which, in combination with conductive gels, can provoke cutaneous reactions after prolonged skin contact [121]. The Ag/AgCl electrodes are also prone to damage after repeated use owing to mechanical stress [121].

Following prototype design, validation was the most identified theme. Validation studies accounted for approximately one-third (29/101, 28.7%) of the total included studies. These studies demonstrated that smart shirts were largely valid in determining cardiorespiratory parameters such as HR and respiratory rate (RR), but showed variable validity when measuring parameters such as energy expenditure (EE), minute ventilation (V_E_), and tidal volume (V_T_) [28,41,107,115,117]. Notably, although maximal oxygen consumption was assessed for reliability in one study, its validity was not evaluated [115]. At the current stage, smart shirts can be considered mostly valid for the measurement of certain physiological parameters under conditions of rest and submaximal activities with variable results at maximal work rate [62,63,74,93,107,115,117]. When comparing the number of prototype design and validation studies, it is evident that a large discrepancy exists. The volume of systematic validation research needs to be increased to meet the rate of prototype design studies being published. This is of importance as these wearables are marketed for use by clinicians and researchers who require valid and reliable measures. Moreover, owing to the fluid state of the software used in these devices, which can receive periodic updates, it is important to validate the new algorithm.

The third theme of study identified by this review was observational research, which comprised 20.8% (21/101) of the total studies. These studies employed a variety of smart shirts in a range of populations, including healthy and clinical as well as specific population subsets such as medical personnel, employees, and emergency operators. A total of 10 distinct smart shirts were utilized, with Hexoskin being the most prevalent. These studies used the data collected by smart shirts for various purposes, such as quantifying stress through heart rate variability (HRV) data in medical personnel and identifying early physiological markers that preceded self-injurious behavior in individuals with intellectual disabilities [35,82,96]. More observational research such as these is required to explore the implementation of smart shirts in live settings and make practical use of the data collected through its processing and analysis. This is the next obvious step in the progression of wearables research.

Finally, only one scoping review was identified in this study, focusing solely on the use of smart textiles for physiological monitoring. As presented in the Results section, this review was restricted to the use of smart textiles for ECG monitoring in cardiac patients.

### Gaps in the Literature

As alluded to in the Principal Findings section, there is a considerable lag between the publication of prototype design and validation studies. Within the area of prototype design, there remains a crucial need to develop sensing technology to further optimize sensor positioning, increase detection sensitivity, and improve the signal-to-noise ratio to assist in moving wearables past the initial prototype stage. In addition, the refinement of algorithms is needed to reduce the risk of overestimating or underestimating values. Furthermore, the diversification of the populations used in validating the prototypes is also needed. Currently, the majority of validation studies include healthy or clinical adults, whereas studies focusing on pediatric populations and specific population subsets such elite athletes and emergency operators are lacking.

Observational research is also largely underrepresented. To understand the capabilities of smart shirts outside of the laboratory, more studies of this nature need to be conducted using a variety of population samples in various settings. These studies should investigate the use of the collected data for meaningful analysis as one issue challenging wearable technology is the translation of its data to create clinically actionable insights. Such insights could take the form of physiological data being collated and reported to a health practitioner on a periodic basis to ensure the optimal management of an outpatient or the implementation of warning signals sent to the patient or health practitioner when the collected data reaches a particular threshold. Although in the sporting realm, teams have begun employing data scientists to disseminate the data into usable metrics, this is largely absent in the clinical sphere. Moving forward, observational studies would be best conducted using a multidisciplinary approach whereby researchers collaborate with the clinicians or professionals expected to implement this technology outside of the laboratory.

### Limitations

This review was limited to publications written in English, which may have excluded key studies published in other languages. This review also focused solely on physiological parameters measured by smart shirts and did not report on any other parameters concerning activity or biomechanics. In addition, the screening, inclusion/exclusion, and data charting stages of this review were conducted by 1 investigator (HK), which could have reduced the likelihood that all relevant studies were identified in the review. The use of 1 investigator may have also resulted in some reviewer bias.

### Comparisons With Previous Work

To the authors’ knowledge, this scoping review is the first to systematically map the scientific body of literature surrounding the use of smart textiles in the form of smart shirts for monitoring physiological parameters across all populations. Only one other review that was included in the results investigated the use of smart shirts for monitoring a single physiological parameter [112]. The inclusion criteria of the aforementioned review were limited to smart shirts capable of monitoring ECG signals in cardiac patients, whereas this scoping review included all physiological parameters and population types.

### Conclusions

With the persistent challenges confronting health systems globally and the rising health and safety demands of athletes and emergency operators, smart textiles present themselves as a contributor to a possible solution. This scoping review systematically surveyed the existing body of scientific literature pertaining to smart textiles in the form of smart shirts for the monitoring of physiological parameters. Through this review, it was identified that the majority of studies surrounding smart textiles were dedicated to prototype design, whereas validation and observational studies lagged behind considerably. Although smart shirts have been proven to be valid and reliable in the monitoring of some physiological parameters such as HR, HRV, and RR, results were variable for other parameters such as V_E_, V_T_, and EE, suggesting a continued need for their systematic validation. Although innovations such as these offer vast potential, it is important to ensure their validity and reliability through careful evaluation before their widespread adoption. To unlock the potential of smart textiles, there is also a need for more observational investigation in collaboration with the professionals expected to implement the technology outside of the laboratory.

## Appendix

Multimedia Appendix 1 Search strategies used for each electronic database searched.

Multimedia Appendix 2 Study selection questionnaire.

Multimedia Appendix 3 Data extraction chart.

Multimedia Appendix 4 Summaries of included validation studies.

Multimedia Appendix 5 Summaries of included observational studies.

## Abbreviations

Ag: silver
AgCl: silver chloride
BT: body temperature
CADTH: Canadian Agency for Drugs and Technologies in Health
ECG: electrocardiograph
EE: energy expenditure
EMBASE: Excerpta Medica database
HR: heart rate
HRV: heart rate variability
JBI: Joanna Briggs Institute
MEDLINE: Medical Literature Analysis and Retrieval System Online
PRISMA: Preferred Reporting Items for Systematic Reviews and Meta-analysis
RR: respiratory rate
sEMG: surface electromyography
SpO_2_: blood oxygen saturation
VE: minute ventilation
VT: tidal volume
