# Supplementary material for: Smart Shirts for Monitoring Physiological Parameters: Scoping Review
Source: JMIR Mhealth Uhealth. 2020 May 27;8(5):e18092. doi: 10.2196/18092 (PMC7287746; doi:10.2196/18092)
Supplement: Multimedia Appendix 1 [file mhealth_v8i5e18092_app1.docx]

Multimedia Appendix - Search strategy used for each electronic database searched.

| **Database** | **Date searched** | **Search strategy** | **# of results** |
| --- | --- | --- | --- |
| Ovid MEDLINE | 24/06/2019 | (Astroskin.ti,ab. OR Hexoskin.ti,ab. OR OMsignal.ti,ab. OR wearable technology.ti,ab. OR wearable sensor.ti,ab. OR e-textile*.ti,ab. OR e-skin*.ti,ab. OR electronic textile*.ti,ab. OR electronic skin*.ti,ab. OR textile electrode*.ti,ab.  OR  ((Biometric*.ti,ab. OR biosens*.ti,ab. OR Sensing System*.ti,ab. OR Sensing.ti,ab. OR Sensor.ti,ab. OR Sensors.ti,ab. OR Tracking.ti,ab. OR Telemetry.ti,ab. Or biocollection.ti,ab. or bio-collection.ti,ab. or wearable*.ti,ab. or (physiological and monitor*).ti,ab.) adj3 (Fabric.ti,ab. OR Fabrics.ti,ab. OR Textile.ti,ab. OR Textiles.ti,ab. OR Garment.ti,ab. OR Garments.ti,ab. OR Shirt.ti,ab. OR Shirts.ti,ab. OR Skin.ti,ab. OR Vest.ti,ab. OR Vests.ti,ab.)) )  NOT (exp Animals/ NOT (exp Animals/ AND exp Humans/)) | 2558 |
| CINHAL | 24/06/2019 | (TI Astroskin OR AB Astroskin OR TI Hexoskin OR AB Hexoskin OR TI OMsignal OR AB OMsignal OR TI "wearable technology" OR AB "wearable technology" OR TI "wearable sensor" OR AB "wearable sensor" OR TI e-textile* OR AB e-textile* OR TI e-skin* OR AB e-skin* OR TI "electronic textile*" OR AB "electronic textile*" OR TI "electronic skin*" OR AB "electronic skin*" OR TI "textile electrode*" OR AB "textile electrode*")  OR  (  ((TI Biometric* OR AB Biometric* OR TI biosens* OR AB biosens* OR TI "Sensing System*" OR AB "Sensing System*" OR TI Sensing OR AB Sensing OR TI Sensor OR AB Sensor OR TI Sensors OR AB Sensors OR TI Tracking OR AB Tracking OR TI Telemetry OR AB Telemetry OR TI biocollection OR AB biocollection OR TI bio-collection OR AB bio-collection OR TI wearable* OR AB wearable* OR (TI physiological OR AB physiological AND TI monitor* OR AB monitor*))  N3  (TI Fabric OR AB Fabric OR TI Fabrics OR AB Fabrics OR TI Textile OR AB Textile OR TI Textiles OR AB Textiles OR TI Garment OR AB Garment OR TI Garments OR AB Garments OR TI Shirt OR AB Shirt OR TI Shirts OR AB Shirts OR TI Skin OR AB Skin OR TI Vest OR AB Vest OR TI Vests OR AB Vests)  ) NOT (MH "Animals+" NOT (MH "Animals+" AND MH "Human+")) | 739 |
| EMBASE | 24/06/2019 | Astroskin:ti,ab OR Hexoskin:ti,ab OR OMsignal:ti,ab OR Hexoskin:ti,ab OR ‘wearable technology’:ti,ab OR ‘wearable sensors’:ti,ab OR e-textile*:ti,ab OR e-skin*:ti,ab OR ‘electronic textile*’:ti,ab OR ‘electronic skin*’:ti,ab OR ‘textile electrode*’:ti,ab  OR  ((Biometric* OR Biosens* OR ‘Sensing System*’ OR Sensing OR Sensor OR Sensors OR Tracking OR Telemetry Or Biocollection OR Bio-collection OR wearable*) NEAR/3 (Fabric OR Fabrics OR Textile OR Textiles OR Garment OR Garments OR Shirt OR Shirts OR Skin OR Vest OR Vests)):ti,ab  OR (physiolog* NEAR/3 monitor* NEAR/3 (Fabric OR Fabrics OR Textile OR Textiles OR Garment OR Garments OR Shirt OR Shirts OR Skin OR Vest OR Vests)):ti,ab | 3,262  (MEDLINE – 1253  EMBASE – 991  Both – 955) |
| Scopus | 24/06/2019 | (TITLE-ABS(astroskin OR hexoskin OR omsignal OR "wearable technology" OR "wearable sensor" OR e-textile OR e-skin* OR "electronic textile*" OR "electronic skin*" OR "textile electrode*")   OR TITLE-ABS ((biometric* OR biosens* OR sensing OR sensor OR sensors OR tracking OR telemetry OR  biocollection OR "Bio-collection" OR wearable* OR (physiological  AND  monitor*)) W/3 (fabric OR fabrics OR textile OR textiles OR garment OR garments OR shirt OR shirts OR skin OR vest OR vests)))  AND  TITLE-ABS (“breathing rate” OR “respiratory rate” OR respiration OR “minute ventilation” OR ventilation OR “breathing frequency” OR VO2max OR VO2peak OR activity OR cadence OR steps OR “energy expenditure” OR workload OR stress OR sleep) | 4,295 |
| Sport Discus | 24/06/2019 | (TI Astroskin OR AB Astroskin OR TI Hexoskin OR AB Hexoskin OR TI OMsignal OR AB OMsignal OR TI "wearable technology" OR AB "wearable technology" OR TI "wearable sensor" OR AB "wearable sensor" OR TI e-textile* OR AB e-textile* OR TI e-skin* OR AB e-skin* OR TI "electronic textile*" OR AB "electronic textile*" OR TI "electronic skin*" OR AB "electronic skin*" OR TI "textile electrode*" OR AB "textile electrode*")  OR  (  ((TI Biometric* OR AB Biometric* OR TI biosens* OR AB biosens* OR TI "Sensing System*" OR AB "Sensing System*" OR TI Sensing OR AB Sensing OR TI Sensor OR AB Sensor OR TI Sensors OR AB Sensors OR TI Tracking OR AB Tracking OR TI Telemetry OR AB Telemetry OR TI biocollection OR AB biocollection OR TI bio-collection OR AB bio-collection OR TI wearable* OR AB wearable* OR (TI physiological OR AB physiological AND TI monitor* OR AB monitor*))  N3  (TI Fabric OR AB Fabric OR TI Fabrics OR AB Fabrics OR TI Textile OR AB Textile OR TI Textiles OR AB Textiles OR TI Garment OR AB Garment OR TI Garments OR AB Garments OR TI Shirt OR AB Shirt OR TI Shirts OR AB Shirts OR TI Skin OR AB Skin OR TI Vest OR AB Vest OR TI Vests OR AB Vests)  ) NOT (MH "Animals+" NOT (MH "Animals+" AND MH "Human+")) | 283 |

This is a Multimedia Appendix to a full manuscript published in the J Med Internet Res. For full copyright and citation information see http://dx.doi.org/10.2196/jmir.18092
