# Supplementary material for: Smart Shirts for Monitoring Physiological Parameters: Scoping Review
Source: JMIR Mhealth Uhealth. 2020 May 27;8(5):e18092. doi: 10.2196/18092 (PMC7287746; doi:10.2196/18092)
Supplement: Multimedia Appendix 2 [file mhealth_v8i5e18092_app2.docx]

Multimedia Appendix - Study selection questionnaire.

| **Questions based on eligibility criteria.** | **Yes** | **No** |
| --- | --- | --- |
| Does the study present or use a smart textile in the form of a shirt? |  |  |
| Does the presented/used smart textile monitor physiological parameters? |  |  |
| Does the presented/used smart textile have integrated sensors? |  |  |
| Are all physiological sensors required for the smart textile system integrated into the shirt? |  |  |
| Does the study focus on the smart textile as an integrated unit? |  |  |
| Does the study meet inclusion criteria? |  |  |

This is a Multimedia Appendix to a full manuscript published in the J Med Internet Res. For full copyright and citation information see http://dx.doi.org/10.2196/jmir.18092
