# Supplementary material for: Smart Shirts for Monitoring Physiological Parameters: Scoping Review
Source: JMIR Mhealth Uhealth. 2020 May 27;8(5):e18092. doi: 10.2196/18092 (PMC7287746; doi:10.2196/18092)
Supplement: Multimedia Appendix 3 [file mhealth_v8i5e18092_app3.docx]

Multimedia Appendix - Data extraction chart.

| **Study Details and Characteristics** | |
| --- | --- |
| **Study Citation Details** | |
| Author (s) |  |
| Year of Publication |  |
| Reference Type |  |
| Title |  |
| Country of Origin |  |
| Study Design |  |
| **Sample** | |
| Setting |  |
| Study Population |  |
| Size |  |
| **Participants** | |
| Mean Age |  |
| Gender – Male / Female |  |
| **Details Extracted from Study** | |
| Study Aim/s |  |
| Methodology Adopted |  |
| Type of Smart Shirt Used (Brand) |  |
| Types of Signals Acquired – Sensors Used (Location of sensor) |  |
| Comparator (If Applicable) |  |
| Outcomes Measured |  |
| Key Findings |  |
| Area of Application (ex. Sport, Clinical, Occupational etc.) |  |

This is a Multimedia Appendix to a full manuscript published in the J Med Internet Res. For full copyright and citation information see http://dx.doi.org/10.2196/jmir.18092
