# Supplementary material for: Smart Shirts for Monitoring Physiological Parameters: Scoping Review
Source: JMIR Mhealth Uhealth. 2020 May 27;8(5):e18092. doi: 10.2196/18092 (PMC7287746; doi:10.2196/18092)
Supplement: Multimedia Appendix 4 [file mhealth_v8i5e18092_app4.docx]

Multimedia Appendix - Summaries of included validation studies.

| **Author**  **Year**  **Country** | **Type of Smart Shirt** | **Study Participants** | **Physiological Metrics Validated** | **Reference Technology** | **Main Findings** |
| --- | --- | --- | --- | --- | --- |
| Hong et al.  2009  South Korea  [119] | BioShirt | Healthy adults | HRV | ECG (leads not specified) | ECG signal detection and transmission capability are similar to conventional ECG. |
| Romagnoli et al.  2014  Spain  [62] | GOW System | Clinical (cardiac patients) | HRV | 12-lead ECG | Substantial motion artefacts during exercise resulted in measurement errors too great compared to 12-lead ECG to be considered a clinical tool for HRV data. |
| Skobel et al.  2014  Germany  [63] | HeartCycle’s Guided Exercise System (GEx) | Clinical (cardiac patients) | HR, arrhythmia detection, RR | Metabolic system (MS) | Acceptable comparability between GEx and reference technology.  GEx can accurately report RR, HR, and ECG data during exercise in cardiac patients. |
| Banerjee et al.  2018  USA  [106] | Hexoskin (HxS) | Clinical (dementia) | HR, RR, V_E_ | Polar HR Monitor (Polar HRM) and MS | HR and RR demonstrated strong positive correlation against reference technology.  V_E_ demonstrated weak positive correlation. |
| Elliot et al.  2019  New Zealand  [115] | HxS | Elite cyclists | HR, RR, V_E_, VO_2max_ | 3-lead ECG, Polar HRM and MS | HxS HR data aligned well with the reference data for all work rate stages.  RR demonstrated good validity from rest through to 75% max work rate (Wmax).  Large discrepancies for V_E_ at all stages of work except for 25% Wmax where it was moderate. |
| Cherif et al.  2018  France  [107] | HxS | Healthy adults | HR, RR, V_E_ | 12-lead ECG and MS | Hxs revealed high correlation coefficients (ICC and CCC) for HR and RR. V_E_ demonstrated variability and weaker correlation coefficients. |
| Montes  2015  USA  [65] | HxS | Healthy adults | HR, RR, EE | Polar HRM, Fitbit Flex and MS | Both HR and RR data were valid and reliable at all walking speeds (1.5 mph, 2.5 mph, 3.5 mph).  V_E_ was reliable but not valid at 1.5 mph and 2.5 mph but neither valid nor reliable at 3.5 mph. |
| Montes et al.  2018  USA  [109] | HxS | Healthy adults | HR, RR, EE | Polar HRM and MS | RR values highly correlated at 1.5 mph and 2.5 mph but demonstrated variations at 3.5 mph.  HR significantly underestimated for all but one minute’s values.  EE had no stages correlated. |
| Montoye et al.  2017  USA  [93] | HxS | Healthy adults | HR, RR, V_T_, EE | MS, Pulse oximeter and Fitbit Charge | Low mean absolute percent error (MAPE) for HxS and Fitbit HR measurments. Both devices had high MAPE values for EE, RR and V_T_. |
| Smith et al.  2019  USA  [117] | HxS | Healthy adults | HR, RR, V_E_, V_T_ | 12-lead ECG and MS | HR and RR revealed high correlation coefficients at rest, submaximal and maximal work.  V_T_ and V_E_ had best agreement at submaximal exercise with larger discrepancies at rest and maximal exercise levels. |
| Tan & Yong  2018  Malaysia  [111] | HxS | Badminton athletes | HR | Samsung Health optical sensor | No significant differences observed between HxS and comparator during a game of badminton. |
| Tanner et al.  2015  USA  [73] | HxS | Healthy adults | HR, RR, V_E_, EE | Polar HRM and MS | V_E_, EE and HR data was not significantly correlated to comparator during trail hiking.  RR was significantly correlated at all time points. |
| Villar et al.  2015  Canada  [74] | HxS | Healthy adults | HR, RR, V_E_, V_T_ | ECG (leads not specified) and MS | No significant differences between HR and RR with respective comparators. HxS produced valid and consistent data for RR, V_T_, V_E_ and HR. |
| Clarenbach et al.  2005  Switzerland  [28] | LifeShirt | Clinical (COPD, CHF) and healthy adults | Respiratory cycle duration, V_E_, V_T_ | Pneumotachograph | No significant bias of respiratory cycle time, V_T_ and V_E_ reported. Agreement between the two methods was similar for clinical and healthy participants. |
| Heilman & Porges  2007  USA  [41] | LifeShirt | Healthy adults | RR-intervals | ECG (leads not specified) | LifeShirt is both accurate in the detection of R-waves and the timing of R-R intervals during baseline and exercise conditions in adults. |
| Kent et al.  2009  UK  [45] | LifeShirt | Healthy adults | HR, RR, expiratory time | Polar Tester and pneumotachograph | Agreement between LifeShirt and comparator is acceptable for constant and incremental work rate exercise.  Reliability was similar between LifeShirt and comparator. |
| Chetelat et al.  2015  Switzerland  [72] | LTMS-S System | Healthy adults | HR, RR, SpO_2_, BT | Polar HRM, MS, Pulse oximeter and ingestible temperature capsule | LTMS-S ECG is comparable to all comparators.  Results obtained for RR were slightly below the 95% to be considered equivalent.  SpO_2_ sensor is within the accuracy recommended by ISO 80601-2-61 standards.  Body temperature correlation analysis showed good agreement. |
| Di Rienzo et al.  2013  Italy  [53] | Maglietta Interattiva Computerizzata (MagIC) | Clinical (cardiac patients) | ECG signals | ECG (leads not specified) | MagIC allowed for correct identification of rhythm type in 92.5% of participants. Estimation of PQ interval and QRS duration similar to reference ECG.  MagIC displayed good performance in detecting arrhythmias. No difference observed in estimation of R-R interval between the two methods. |
| Di Rienzo et al.  2005  Italy  [29] | MagIC | - Clinical (cardiac patients) and healthy adults | ECG signals | 3-lead ECG | MagIC provided readable ECG signals for more than 99% and 97% of participants when in supine or on cycle ergometer respectively.  MagIC correctly estimated R-R intervals during exercise. |
| Curone et al.  2010  Italy  [46] | ProeTEX (inner garment) | Healthy adults | HR, RR, SpO2, BT | SuperMon HRM, spirometer and rectal probe | HR and RR were considered acceptable at trails carried out at 25℃ and 35℃. Shirt malfunction detected at 45℃ due to excessive sweating. |
| Magenes et al.  2011  Italy  [48] | ProeTEX (inner garment) | Healthy adults (professional firefighters) | HR, RR, BT | ECG (leads not specified), spirometer and rectal probe | The system demonstrated to be reliable even when employed for extended periods (more than 1 h) in 25℃ to 30℃ environments and up to 30 mins at 45℃. |
| Yu et al.  2016  Germany  [81] | Prototype 1 | Healthy Adults | ECG signals | 12-lead Holter ECG | Prototype demonstrated very high accuracy for P, Q, R, S, T wave detection when tested in multiple scenarios (supine, sitting, walking). |
| Cleland et al.  2012  UK  [52] | Prototype 2 | Healthy adults | HR | 1-lead ECG | Results indicate the prototype did not perform accurately whilst performing tasks involving movement of the trunk and limbs. |
| Lo Presti et al.  2017  Italy  [92] | Prototype 3 | Healthy adults | RR, V_T,_ inspiratory/expiratory times, total respiratory duration | 8-camera Optoelectronic Motion Capture System (MoCap – Smart D) | RR demonstrated high accuracy. V_T_ monitoring demonstrated high average percentage differences in respect to comparator. |
| Massaroni et al.  2018  Italy  [108] | Prototype 4 | Healthy adults | RR, V_T,_ inspiratory/expiratory times, total respiratory duration | (MoCap – Smart D) | Good agreement between the two systems on respiratory periods, frequency and V_T_. Prototype demonstrated good performance in monitoring thoraco-abdominal pattern and its variations. |
| Presti et al.  2018  Italy  [110] | Prototype 5 | Healthy adults | RR, V_T_, **∆V_T_** | (MoCap – Smart D) | Prototype demonstrated good performance in RR monitoring.  High average percentage differences demonstrated for V_T_ in respect to comparator.  Bland-Altman analysis demonstrated weak capabilities of prototype in breath-by-breath analysis of changes in V_T_ in females. |
| Bourdon et al.  2005  France  [27] | Wealthy | Clinical (cardiac patients) and healthy adults | HR, RR, V_T_ | 5-lead ECG and vitaport Polygraphic recorder | Wealthy system provided ECG and respiratory signals as good as comparator in healthy participants.  The Wealthy signal is superior to comparator with heavy sweating.  In clinical participants the Wealthy system is not consistently different than comparator. |
| Reali et al.  2019  Italy  [116] | WWS Smartex | Healthy adults | HRV | 1-lead EC and photoplethysmography (research-grade wristwatch) | HRV signals collected from WWS and comparator found to have similar mean, SD, power spectral density and band power in low and high frequency.  WWS shirt presented very low average MAPE. |
| Dolezal et al.  2014  USA  [61] | Zephyr BioHarness System | Healthy adults | HR | 12-lead ECG | The shirt provided a valid measurement of HR during simulated firefighting duties.  The smallest difference was seen during search and crawl task while greatest discrepancy was during ascending/descending stairs. |

This is a Multimedia Appendix to a full manuscript published in the J Med Internet Res. For full copyright and citation information see http://dx.doi.org/10.2196/jmir.18092
