# Supplementary material for: Smart Shirts for Monitoring Physiological Parameters: Scoping Review
Source: JMIR Mhealth Uhealth. 2020 May 27;8(5):e18092. doi: 10.2196/18092 (PMC7287746; doi:10.2196/18092)
Supplement: Multimedia Appendix 5 [file mhealth_v8i5e18092_app5.docx]

Multimedia Appendix - Summaries of included observational studies.

| **Author**  **Year**  **Country** | **Type of Smart Shirt** | **Aim** | **Population** | **N of Participants** | **Study Setting** | **Physiological Outcome Measures** |
| --- | --- | --- | --- | --- | --- | --- |
| Baig et al.  2017  New Zealand  [94] | Hexoskin (HxS) | To use HxS for real-time monitoring, detection and self-management of T2DM. | Clinical – T2DM | 2 | Controlled and field | RR, V_T_, EE, HR, HRV |
| Baig et al.  2018  New Zealand  [91] | HxS | To use HxS physiological data to assist with the early detection of prediabetes and T2DM. | Clinical – T2DM | 2 | Not specified | RR, V_T_, EE, HR, HRV |
| Omurtag et al.  2018  UK  [82] | HxS | To use HxS’ physiological data to quantify mental workload in the OR. | Surgeons and non-surgeons | 22 | Controlled | RR, HR, HRV |
| Montes et al.  2015  USA  [65] | HxS | To obtain physiological data in pilot study during trail hiking. | Healthy volunteers | 10 | Field: outdoors trail | RR, RR_max_, EE, HR, HR_max_ |
| Slamon et al.  2018  USA  [96] | HxS | To use the HxS to measure physiological data of critical care physicians during live clinical scenarios. | Critical care physicians | 5 | Field: PICU children’s hospital | RR, HR, HRV |
| Thompson et al.  2018  USA  [97] | HxS | To use the HxS to assess differences in occupational physical activity among workers in active and sedentary environments. | Brewery and office employees | 94 | Field: brewery and office | EE, HR, %HR_max_, |
| Webster et al.  2017  USA  [85] | HxS | To use the HxS to predict future cognitive states in mood, motivation, or behavioural context. | Office employees | 5 | Field: offices (not specified) | RR, V_T_, V_E_, HR |
| Barrera et al.  2007  Canada  [35] | HxS | To use the HxS’ physiological data to identify patterns of self-injurious behaviour. | Clinical – developmental disabilities | 3 | Controlled | HR |
| Halin et al.  2005  Finland  [23] | LifeShirt | To examine the functionality and reliability of the LifeShirt during daily activities in a hospital OR. | Clinical (endoscopic day patients) and  healthy | 20 (10 clinical, 10 healthy) | Field (OR and free living) | RR, V_T_, ECG signals |
| Di Rienzo et al.  2006  Italy  [30] | MagIC | To monitor vital signs of cardiac inpatients and healthy participants during exercise under gravitational stress. | Clinical (cardiac patients) and healthy | 40 (31 clinical, 9 healthy) | Field (Cardiac Rehabilitation Unit (CRU)) and parabolic flight | HR, R-R intervals, QRS complex |
| Di Rienzo et al.  2014  Italy  [54] | MagIC | To use the MagIC system to derive a 24-hour profile of cardiac time intervals performed in daily life. | Healthy volunteers | 2 | Controlled | Seismocardiogram |
| Mirmohamadsadeghi et al.  2014  Switzerland  [55] | Model SEW | To devise features with clinical relevance to detect sleep apnea using interpretation of ECG and respiration recordings. | Healthy volunteers | 12 | Controlled (hypoxic chamber) and field (free living at various altitudes) | Respiration waveforms, R-peak, respiratory sinus arrhythmia |
| Balsam et al.  2018  Poland  [95] | Nuubo ECG System | To provide rationale of using the Nuubo system in monitoring patients with CVD in different clinical situations. | Clinical - cardiac patients | 160 (120 adults, 40 paediatric) | Field: CRU | HR, ECG signals, arrhythmia |
| Presti et al.  2017  Italy  [84] | Prototype | To evaluate the use of a prototype shirt in subjects undergoing a magnetic resonance imaging. | Healthy volunteers | 2 | Field: MRI machine | RR, Inspiratory/Expiratory Ratio |
| Yu et al.  2018  Germany  [98] | Prototype | To evaluate the use of a prototype shirt in the detection of occasional arrhythmia in a clinical study through 422 hours of recording. | Clinical – cardiac patients | 5 | Field: free living | ECG signals |
| Lanata et al.  2015  Italy  [64] | PSYCHE | To present results gathered from tracking 10 participants with bipolar disorder who exhibited severe symptoms (depression, hypomania, mixed state etc.). | Clinical – bipolar disorder | 10 | Filed: hospital | HR, HRV |
| Valenza et al.  2016  Italy  [76] | PSYCHE | To propose a methodology to predict mood changes in bipolar disorder using heartbeat nonlinear dynamics derived by PSYCHE system. | Clinical – bipolar or cyclothymic disorder | 14 | Field: free living | RR, HR, HRV |
| Valenza et al.  2014  Italy  [56] | PSYCHE | To propose the use of PSYCHE to acquire ECG, respirogram information to detect a pattern of physiological parameters to support diagnosis. | Clinical - bipolar | 8 | Field: free living | HRV |
| Migliorini et al.  2012  Italy  [49] | Smartex | To define physiological parameters and vitals that may relate to the mood and mental status in patients with bipolar disorder. | Clinical (bipolar) and healthy volunteers | 9 (1 clinical, 8 healthy) | Controlled | HR, HRV, R-R intervals |
| Palix et al.  2017  Switzerland  [83] | Smartex | To investigate the use of HRV as a physiological marker that precedes increase in behavioural excitation of intellectually disabled individuals. | Clinical – intellectual disability | 13 | Field: special care facility and free living | HRV |
| Paiva et al.  2016  Portugal  [75] | VitalJacket | To use the VitalJacket in assessing morphological changes in ECG signals induced by acute stress. | Healthy - firefighters | 6 | Controlled | R-R interval, QT and ST intervals |

This is a Multimedia Appendix to a full manuscript published in the J Med Internet Res. For full copyright and citation information see http://dx.doi.org/10.2196/jmir.18092
